# ZJU index as a predictive biomarker of gestational diabetes mellitus: a prospective cohort analysis

**DOI:** 10.3389/fnut.2025.1570771

**Published:** 2025-06-13

**Authors:** Ziyi Xu, Xuewei Li, Hui Wang, Liuyang Xu, Changhui Li

**Affiliations:** ^1^College of Traditional Chinese Medicine, Changchun University of Chinese Medicine, Changchun, China; ^2^The Affiliated Hospital of Changchun University of Chinese Medicine, Changchun, China

**Keywords:** ZJU, gestational diabetes mellitus, liver enzyme, early pregnancy, lipid index

## Abstract

**Background:**

The ZJU index, incorporating fasting plasma glucose (FPG), lipid profiles, liver enzymes, and body mass index (BMI), serves as a multidimensional tool for assessing metabolic dysregulation. This prospective investigation examined first-trimester ZJU index associations with both gestational diabetes mellitus (GDM) risk and nonalcoholic fatty liver disease (NAFLD) during pregnancy, while simultaneously evaluating the relationships between lipid profiles, liver enzymes, and GDM development.

**Methods:**

We conducted analyses using multivariable logistic regression and restricted cubic splines (RCS) to assess associations of the ZJU index, liver enzymes, and blood lipids with GDM, as well as the association between the ZJU index and NAFLD. Subgroup analyses were conducted to evaluate the correlation between the ZJU index (stratified by age and reproductive history) and GDM. The receiver operating characteristic (ROC) curve assessed the ZJU index’s predictive power. The robustness of the findings was verified via sensitivity analyses.

**Results:**

In the multivariable regression model, the ZJU index showed a significant positive association with GDM, after adjusting for confounders [OR = 1.22, 95% CI (1.13–1.32)]. The RCS analysis revealed a linear dose-response relationship between the ZJU index and GDM. The area under the curve (AUC) for the ZJU index was 0.802, indicating a high predictive ability for GDM. Associations between the ZJU index and GDM remained consistent across subgroups and sensitivity analyses.

**Conclusion:**

The ZJU index is closely associated with GDM prevalence.

## 1 Introduction

Gestational diabetes mellitus (GDM), characterized by hyperglycemia first detected during pregnancy, is the most common gestational metabolic disorder, affecting 14% of pregnancies and posing a significant health burden worldwide (1, 2). The etiology of GDM is multifactorial, involving complex interactions among genetic, epigenetic, and environmental elements. Recognized risk factors include maternal obesity, advanced maternal age, multiple pregnancies, and excessive gestational weight gain (3, 4). GDM substantially increases the risk of perinatal complications such as pre-eclampsia and macrosomia, while also significantly raising the long-term risk of metabolic disorders like obesity, cardiovascular disease (CVD), and type 2 diabetes mellitus (T2DM) for mothers and their offspring (5–7). Recent research indicates that managing GDM in the early pregnancy (before 20 weeks) can mitigate gestational and postnatal complications, improving long-term health outcomes (8–11). Nevertheless, traditional mono-biomarkers for early GDM screening exhibit limitations in sensitivity and specificity (12), posing obstacles in precise clinical risk assessment and underscoring the need for efficient, cost-effective screening innovations.

Metabolic disorders during early pregnancy have been linked to the onset of GDM (12). Distinctive biomarker changes, including elevated liver enzymes [e.g., alanine aminotransferase (ALT) and aspartate aminotransferase (AST)] and dyslipidemia [e.g., heightened triglyceride (TG)], have been documented in GDM patients (13–16). However, studies across diverse populations exhibit significant heterogeneity in the associations between liver enzymes, lipid profiles, and GDM risk. Notably, NAFLD is a pathological manifestation of the metabolic syndrome at the hepatic level and is strongly associated with the risk of GDM (17, 18). Nonetheless, the feasibility and safety of using imaging to screen NAFLD during pregnancy are limited. Thus, clarifying the independent associations of liver enzymes and lipid markers with GDM, and developing reliable predictive biomarkers for NAFLD during pregnancy, hold substantial clinical importance.

The ZJU index, a novel NAFLD biomarker developed in China, combines fasting plasma glucose (FPG), TG, ALT/AST ratio, and body mass index (BMI) to assess comprehensive metabolic status (19). Compared with traditional single biomarkers, it comprehensively captures metabolic dysfunction. It has demonstrated higher predictive efficacy for NAFLD in numerous studies and is strongly associated with T2DM (19–21). However, its application during pregnancy demands exploration. It is essential to recognize that both GDM and T2DM derive from similar underlying mechanisms involving insulin resistance and glucose-lipid metabolism disorders. Furthermore, lipid anomalies during early pregnancy precede clear elevations in glycemic levels (22). Therefore, the relationship between the ZJU index and GDM should be explored further.

Considering the substantial impact of GDM and its associated complications, identifying valid biomarkers for early risk assessment is of significant clinical importance. While the ZJU index has been validated in general populations for NAFLD and diabetes risk, its predictive value during the early stages of pregnancy for GDM remains unexplored. This study hypothesizes that higher ZJU index values at 10–14 weeks of gestation are linked to an elevated risk of developing GDM.

## 2 Materials and methods

### 2.1 Study design

To investigate the relationship of ZJU with GDM, we used studies from South Korea for a secondary analysis of the data. The prospective cohort study was from Korea, the “fatty liver in pregnancy” registry (NCT02276144) (23).

### 2.2 Data source

Prospective studies from Korea were sourced from a publication published in PLoS One, volume 14, issue 8, article number e0221400 (2019). In accordance with the stipulations of the Creative Commons Attribution License, the material in question is made available for use, distribution and replication in any format, free of charge, on the condition that the source and author are duly acknowledged (23).

### 2.3 Study participants

A total of 663 pregnant women with singleton pregnancies at ≤14 weeks of gestation were enrolled from Seoul National University Boramae Medical Center (affiliated with the Seoul Metropolitan Government) and Seoul Women’s Hospital in Incheon. Data collection was conducted as part of the ongoing “Fatty Liver in Pregnancy” registry between November 2014 and July 2016 (ClinicalTrials.gov, Registration No. NCT 02276144). Prior to registration, all subjects were required to sign an informed consent form. The studies involving humans were approved by the Institutional Review Board of the Seoul National University Boramae Medical Center and the Public Institutional Review Board of the Korean Ministry of Health and Welfare. No further ethical review was required for our secondary analysis. The original study was conducted in accordance with the principles set forth in the Declaration of Helsinki, and our secondary analysis was performed in compliance with the STROBE guidelines, as applicable.

**Figure 1:**
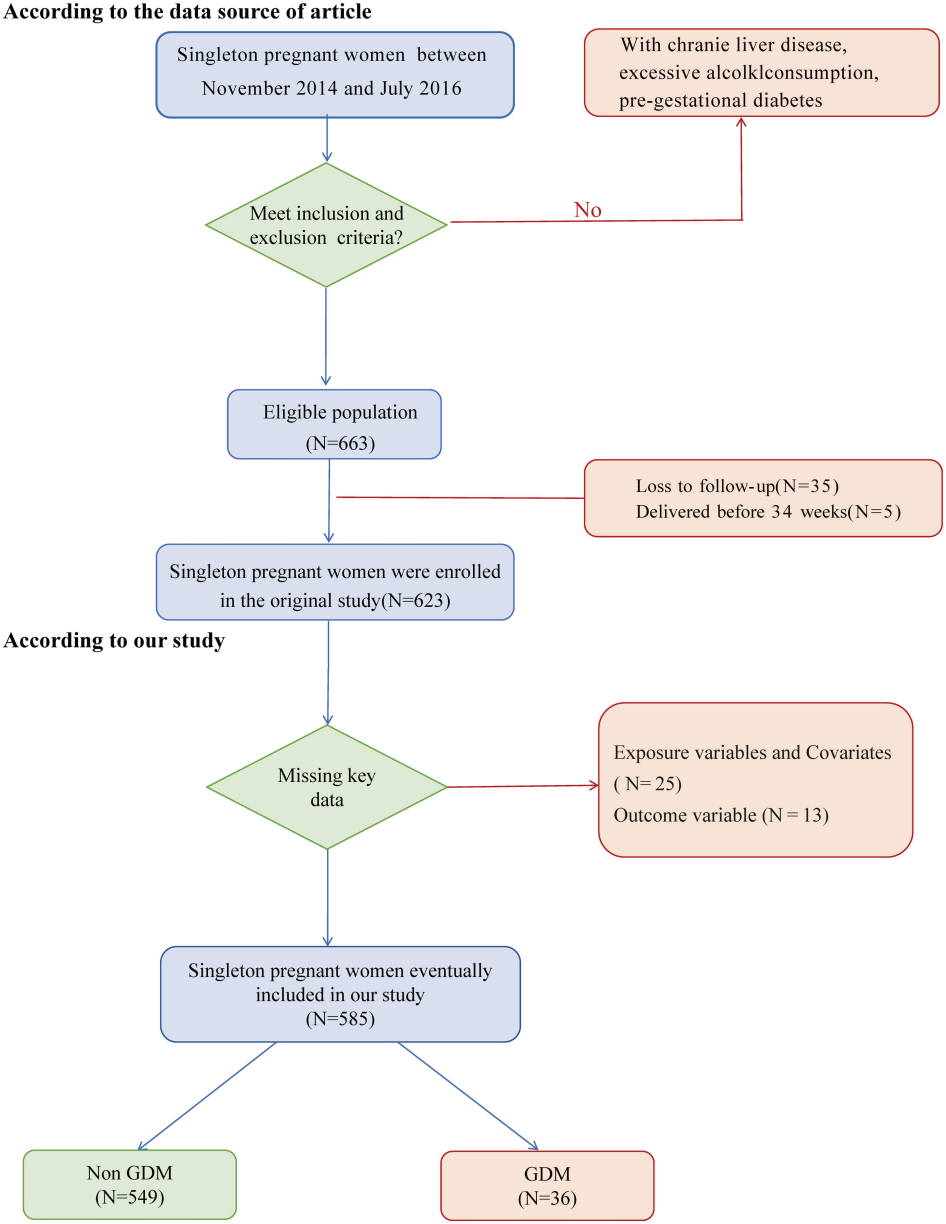
Flowchart of the study design.

Between November 2014 and July 2016, a total of 663 pregnant women without chronic liver disease, a history of alcohol abuse, or pregestational diabetes were enrolled, all of whom underwent liver ultrasonography at 10–14 weeks of gestation. After excluding individuals lost to follow-up (*n* = 35) and those who delivered before 34 weeks of gestation (*n* = 5), a final analytic cohort of 623 participants was included in the data analysis (17). The study initially encompassed 585 singleton pregnancies. Of these, 38 cases were excluded due to incomplete key datasets, including 25 cases with missing lipid profiles [TG, high-density lipoprotein (HDL)], incomplete liver function tests (ALT, AST), BMI and metabolic markers (FPG, insulin), as well as 13 cases with lack of diagnostic data for GDM. The comprehensive participant screening process is systematically delineated in Figure 1.

### 2.4 Measurement of GDM and NAFLD

In accordance with guidelines from the American College of Obstetricians and Gynecologists (ACOG), all participants underwent a two-step screening protocol for GDM at 24–28 weeks of gestation (24). The first step involved a 50-g oral glucose challenge test (GCT) administered in a non-fasting state, with capillary blood glucose measured 1 h after glucose ingestion. A GCT result of ≥7.8 mmol/L was considered positive. For women with a positive GCT, a subsequent 100-g oral glucose tolerance test (OGTT) was performed. GDM was diagnosed when two or more of the following OGTT thresholds were met: fasting plasma glucose ≥5.3 mmol/L, 1-h glucose ≥10.0 mmol/L, 2-h glucose ≥8.6 mmol/L, or 3-h glucose ≥7.8 mmol/L. The GDM (dichotomous: 0 = non-GDM, 1 = GDM) is the outcome variable.

The presence of NAFLD is defined as a bright echo pattern in the liver detected by ultrasound.

### 2.5 Assessment of covariates

General clinical and demographic information, including maternal age, parity, and pre-gestational height and weight, was collected using the validated cut-annoyed-guilty-eye questionnaire (25). BMI was calculated as the body weight divided by the standing height squared.

Venous blood samples were collected from participants between 10 and 14 weeks of gestation following an overnight fast of at least 8 h. All samples underwent centrifugation, aliquotation, and storage at −70°C for subsequent analysis of hematological parameters. FPG, routine lipid profiles, and hepatic enzyme concentrations were quantified using enzymatic assays on a Roche/Hitachi 911 chemistry analyzer (Roche Diagnostics, Indianapolis, IN, United States), with glucose measured via the hexokinase method. Insulin concentrations were assayed using a single-batch immunoradiometric kit (INS-IRMA; DIAsource ImmunoAssays, Louvain-la-Neuve, Belgium), and adiponectin levels were determined by enzyme-linked immunosorbent assay (ELISA; R&D Systems, Minneapolis, MN, United States).

Covariates were selected using a hybrid analytical framework that synergized data-driven techniques (bidirectional stepwise regression) with theory-informed approaches incorporating directed acyclic graphs (DAGs), existing evidence from literature, and clinical expertise (26). The final adjusted model included three covariates: maternal age, nulliparity, and adiponectin levels.

### 2.6 Measurement of ZJU

Venous blood samples were collected from participants between 10 and 14 weeks of gestation, following an overnight fast of at least 8 h. All samples were centrifuged and stored at −70°C for subsequent analysis of hematological markers, including FPG (mmol/L), ALT (U/L), AST (U/L), TG (mg/dL) (19).

$$\begin{array}{l} \mathrm{ZJU}\;\text{index}=\mathrm{FPG}\;(\text{mmol}/L)+\mathrm{BMI}\;(\mathrm{kg}/m^{2})+3^{*}\mathrm{ALT}\;(U/L)/\\ \mathrm{AST}\;(U/L)\;\text{ratio}\;(+2\;\text{if female})+\mathrm{TG}\;(\text{mmol}/L) \end{array}$$

### 2.7 Statistical analysis

Baseline characteristics of the participants were presented according to their GDM status. For managing missing data, we employed listwise deletion, removing any observations with incomplete values from all analyses. The analysis included means and standard deviations for continuous variables, and frequencies and percentages for categorical variables. Group comparisons for continuous variables were performed using the *t*-test or analysis of variance, while chi-square tests were utilized for categorical variables. The Box-Tidwell method was employed to test the linearity of logarithmic variables. Covariates selection was guided by the 10 events per variable principle and which adopted a hybrid approach combining data-driven bidirectional stepwise regression and theory-driven methods, including DAGs (Supplementary Figure S1), clinical expertise, and prior literature (26, 27). Multicollinearity among independent variables was assessed using the variance inflation factor (VIF). Multivariable logistic regression and restricted cubic splines (RCS) were used to assess associations of the ZJU index with liver enzymes, blood lipids, and GDM, as well as the association between the ZJU index and NAFLD.

The three models were estimated in order to perform the requisite tests. In the study, model1 no adjustments were made for potential confounding variables; model 2 was adjusted for age and nulliparity; and model 3 was adjusted for age, nulliparity and adiponectin. Besides, a multivariable logistic regression model was employed to assess the correlation between liver enzyme, blood lipid and GDM. The adjustment factors include: age, nulliparity, and pregnancy BMI. Receiver operating characteristic (ROC) curves were used to assess the predictive accuracy of the GDM, and the Youden index method determined the optimal predicted probability cut-off points. Subgroup analysis methods explored the relationship between the ZJU index and GDM, stratified by age and nulliparity. Guided by clinical insights and prior evidence (17, 28), we conducted four sensitivity analyses to assess the robustness of ZJU index-GDM associations: (1) to address bias, we used 1:1 propensity score matching (PSM) with greedy nearest neighbor matching (caliper = 0.02), matching on age and nulliparity. Post-matching balance was confirmed via standardized mean differences (SMD <0.1). Multivariable logistic regression was applied to matched data; (2) including NAFLD in our fully adjusted model; (3) exclusive inclusion of participants with normal hepatic function (ALT ≤40 U/L, AST ≤40 U/L, GGT ≤50 U/L); (4) application of Firth penalized regression to address potential small-sample bias. As the sample size was entirely dependent on the available data, no formal power calculation was performed, and the analyses were exploratory in nature.

Data processing and analysis were performed using Empower software (www.empowerstats.com; X&Y solutions, Inc., Boston, MA) and R version 4.4.0 (2024-04-24). A < 0.05 *p*-value was considered a statistically significant difference.

## 3 Results

### 3.1 Clinical characteristics of study participants

Table 1 summarizes demographic and clinical characteristics stratified by GDM status. Among 585 participants, 36 (6.2%) developed GDM, with an overall median maternal age of 32 years. Compared to non-GDM counterparts, women with GDM exhibited significantly higher prevalence of NAFLD (*p* < 0.05) and demonstrated distinct metabolic profiles characterized by elevated fasting biomarkers (FPG, insulin, FFA), increased homeostatic model assessment of insulin resistance (HOMA-IR), reduced adiponectin levels, dyslipidemia (elevated TG, decreased HDL), and hepatic dysfunction (abnormal ALT and GGT activities) (all *p* < 0.05).

**Table 1:** Clinical characteristics of study participants.

Characteristic	Without GDM (*N* = 549)	With GDM (*N* = 36)	*p*-value
Age (years)	32.0 ± 3.8	32.6 ± 3.6	0.586
BMI (kg/m^2^)	21.8 ± 3.2	25.8 ± 5.2	<0.001
AST (IU/L)	17.7 ± 8.2	19.7 ± 7.3	0.061
ALT (IU/L)	13.1 ± 9.2	18.8 ± 13.3	0.002
GGT (IU/L)	13.7 ± 8.4	17.7 ± 8.9	0.002
TC (mg/dL)	172.4 ± 26.9	180.2 ± 29.7	0.145
TG (mg/dL)	115.1 ± 41.7	176.1 ± 83.1	<0.001
HDL (mg/dL)	65.3 ± 13.2	58.9 ± 16.5	0.038
LDL (mg/dL)	84.0 ± 21.2	84.0 ± 28.7	0.912
FBG (mg/dL)	76.5 ± 9.0	84.6 ± 15.2	0.001
Insulin (μIU/mL)	9.1 ± 6.2	16.3 ± 8.9	<0.001
FFA (μEq/L)	642.5 ± 268.9	743.6 ± 304.9	0.035
ZJU	31.5 ± 3.7	37.2 ± 5.6	<0.001
Nulliparity			0.97
No	288 (52.5%)	19 (52.8%)	
Yes	261 (47.5%)	17 (47.2%)	
NAFLD			<0.001
No	459 (83.6%)	16 (44.4%)	
Yes	90 (16.4%)	20 (55.6%)	
Adiponectin (ng/mL)	6,297 ± 4,305	2,622 ± 2,156	<0.001
HOMA-IR	1.8 ± 1.7	3.6 ± 2.4	<0.001

BMI, body mass index; TG, triglyceride; TC, total cholesterol; HDL, high-density lipoprotein cholesterol; LDL, low-density lipoprotein cholesterol; FPG, fasting plasma glucose; ALT, alanine Aminotransferase; GGT, gamma-glutamyltransferase; AST, aspartate aminotransferase; FFA, free fatty acid; NAFLD, nonalcoholic fatty liver disease; HOMA-IR, homeostatic model assessment of insulin resistance; GDM, gestational diabetes mellitus. Values were expressed as mean (standard deviation) or *n* (%).

### 3.2 Relationship between ZJU index with GDM and NAFLD

Supplementary Table S1 demonstrated that all variables in the multicollinearity diagnosis had VIF <5, indicating no multicollinearity issues. The Box-Tidwell test in Supplementary Table S2 revealed a linear relationship between the ZJU index and GDM risk (*p* = 0.96), satisfying the linearity assumption for logistic regression.

**Figure 2:**
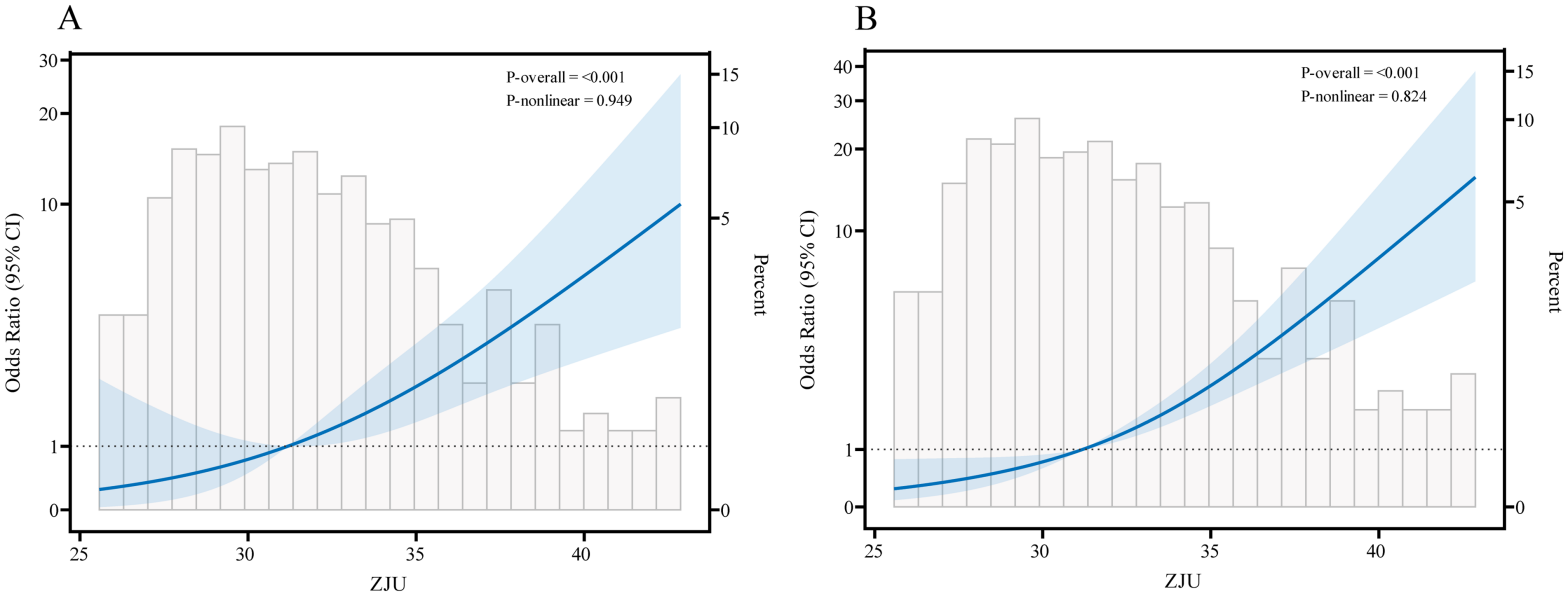
Dose-response relationship between ZJU with GDM and NAFLD. **(A)** Dose-response relationship between ZJU with GDM. **(B)** Dose-response relationship between ZJU with NAFLD.

We evaluated the association between the ZJU index both as a continuous variable and within tertile groups and GDM. Results from Table 2 showed that each 1-unit increase in the ZJU index was associated with a 22% higher GDM risk (OR = 1.22, 95% CI: 1.13–1.32) in Model 3. Tertile-group analysis further revealed a significant trend of increased GDM risk across ZJU index tertiles (*p* for trend <0.05). The RCS analysis confirmed a significant linear association between the ZJU index and GDM risk (*p* nonlinear = 0.949), with the dose-response relationship illustrated in Figure 2.

**Table 2:** Association between ZJU with GDM and NAFLD in multivariable logistic regression.

Variable	OR (95% CI), *p*-value		
	Model 1	Model 2	Model 3
Association between ZJU and GDM			
Continuous			
ZJU	1.30 (1.20, 1.40) < 0.0001	1.30 (1.21, 1.41) < 0.0001	1.22 (1.13, 1.32) < 0.0001
Categories			
Q1 (23.84–29.65)	Ref.	Ref.	Ref.
Q2 (29.65–32.88)	4.13 (0.87, 19.70) 0.0753	4.10 (0.86, 19.57) 0.0768	3.66 (0.75, 17.80) 0.1080
Q3 (32.89–48.61)	14.85 (3.47, 63.48) 0.0003	14.84 (3.47, 63.48) 0.0003	7.70 (1.76, 33.71) 0.0068
*p* for trend	<0.0001	<0.0001	0.0017
Association between ZJU and NAFLD			
Continuous			
ZJU	1.27 (1.20, 1.34) < 0.0001	1.28 (1.21, 1.35) < 0.0001	1.26 (1.19, 1.34) < 0.0001
Categories			
Q1 (23.84–29.65)	Ref.	Ref.	Ref.
Q2 (29.65–32.88)	1.45 (0.72, 2.90) 0.2960	1.46 (0.73, 2.92) 0.2879	1.42 (0.71, 2.85) 0.3250
Q3 (32.89–48.61)	7.34 (4.02, 13.38) < 0.0001	7.43 (4.07, 13.57) < 0.0001	6.35 (3.42, 11.79) < 0.0001
*p* for trend	<0.0001	<0.0001	<0.0001

OR, odds ratio; CI, confidence interval. Model 1: no covariates were adjusted. Model 2: adjusted for age and nulliparity. Model 3: adjusted for age, nulliparity, and adiponectin.

**Figure 3:**
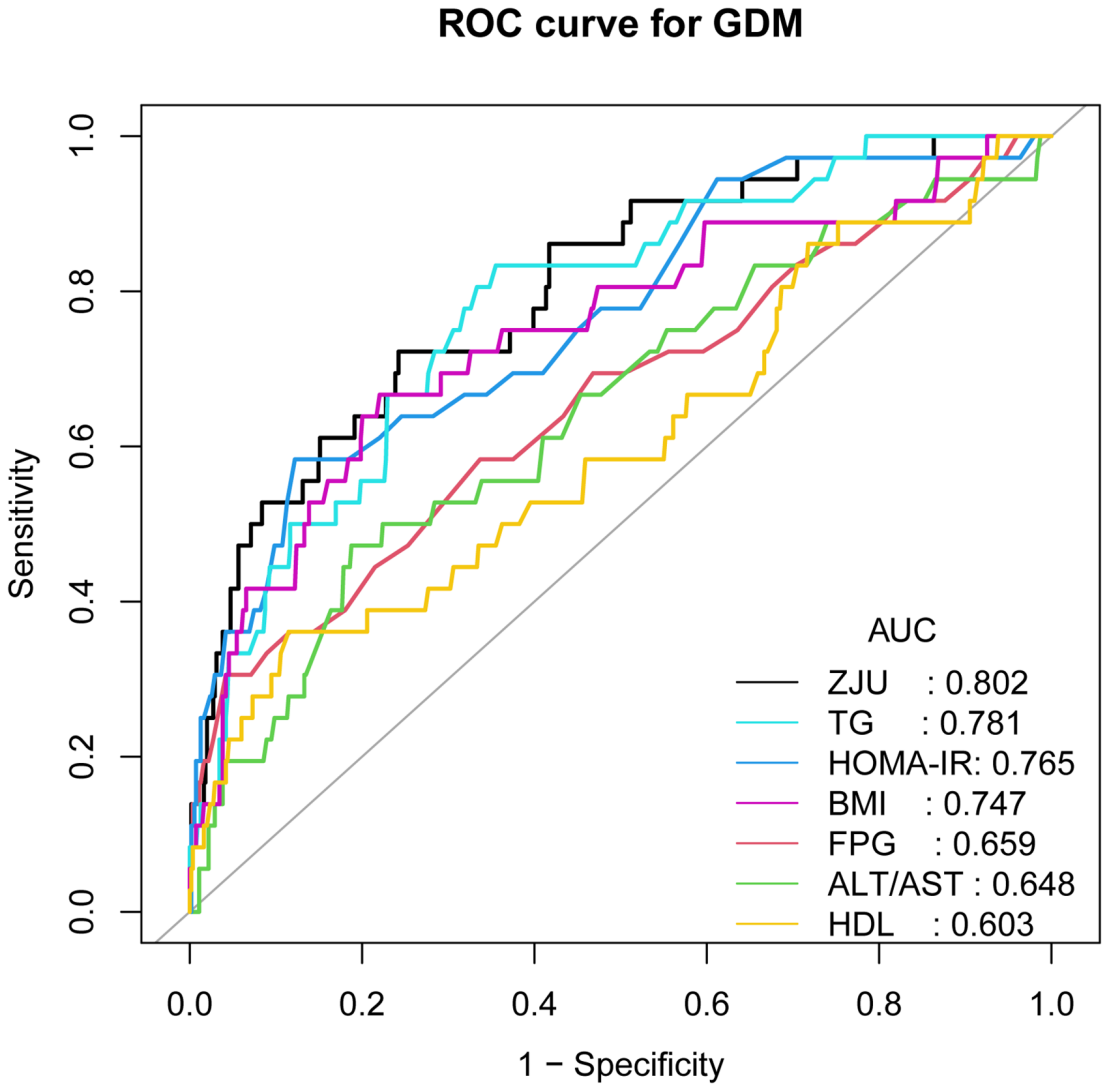
ROC curves for predicting GDM. AUC, area under the curve; BMI, body mass index; TG, triglyceride; HDL, high-density lipoprotein cholesterol; FPG, fasting plasma glucose, ALT, alanine aminotransferase; AST, aspartate aminotransferase; HOMA-IR, homeostatic model assessment of insulin resistance; GDM, gestational diabetes mellitus.

Furthermore, Table 2 also showed a significant positive correlation between the ZJU index and NAFLD after adjusting for the same covariates (OR = 1.26, 95% CI: 1.19–1.34, *p* < 0.0001). In Figure 3, RCS analysis confirmed a linear dose-response relationship, as indicated by *p* nonlinear = 0.824.

### 3.3 Associations between liver enzymes, lipids index and GDM

Table 3 shows the relationship between liver enzymes, blood lipids, and GDM after adjusting for all confounding factors. Single liver enzymes in early pregnancy were not associated with GDM, while ALT / AST showed a significant positive association with GDM [OR 2.80 95% CI (1.04–7.56)]. TG in early pregnancy was associated with GDM risk, with one unit of TG elevation indicating a 2% increase in the risk of GDM [OR 1.02 95% CI (1.01–1.02)]. Early pregnancy HDL and GDM demonstrated a protective relationship [OR 0.96, 95% CI (0.94–0.99)].

**Table 3:** Associations between liver enzymes, lipids index, and GDM.

Variables	OR (95% CI), *p*-value		
	Model 1	Model 2	Model 3
GGT	1.03 (1.01, 1.06) 0.0118	1.03 (1.01, 1.06) 0.0115	1.01 (0.98, 1.05) 0.5920
ALT	1.04 (1.01, 1.06) 0.0018	1.04 (1.02, 1.07) 0.0012	1.03 (1.00, 1.05) 0.0507
AST	1.02 (0.99, 1.05) 0.1754	1.02 (0.99, 1.05) 0.1696	1.01 (0.98, 1.05) 0.3856
ALT/AST	4.22 (1.74, 10.23) 0.0014	4.56 (1.84, 11.27) 0.0010	2.80 (1.04, 7.56) 0.0415
TG	1.02 (1.01, 1.02) < 0.0001	1.02 (1.01, 1.03) < 0.0001	1.02 (1.01, 1.02) < 0.0001
HDL-C	0.96 (0.94, 0.99) 0.0056	0.96 (0.94, 0.99) 0.0053	0.96 (0.94, 0.99) 0.0079
TC	1.01 (1.00, 1.02) 0.0929	1.01 (1.00, 1.02) 0.0950	1.01 (0.99, 1.02) 0.3528
LDL	1.00 (0.98, 1.02) 0.9981	1.00 (0.98, 1.02) 0.9998	0.99 (0.98, 1.01) 0.3305

OR, odds ratio; CI, confidence interval. Model 1: no covariates were adjusted. Model 2: adjusted for age and nulliparity. Model 3: adjusted for age, nulliparity, and pre-pregnancy BMI.

### 3.4 Subgroup analysis

Table 4 shows the results of the subgroup analysis stratified by age, nulliparity. No significant interactions were observed between these variables and the effect of the ZJU index on GDM risk (age: *p*-interaction = 0.931; nulliparity: *p*-interaction = 0.750). The results suggest that the relationship between ZJU and GDM is robust.

**Table 4:** Subgroup analysis of the associations between ZJU and GDM.

Subgroups	OR (95% CI)	*p* for interaction
Age (years)		0.931
<30	1.22 (1.03, 1.43) 0.020	
≥30	1.23 (1.12, 1.35) < 0.001	
Nulliparity		0.750
No	1.25 (1.11, 1.40) < 0.001	
Yes	1.19 (1.06, 1.33) 0.002	

OR, odds ratio; CI, confidence interval. Adjusted for all covariates except for this subgroup of variables (adjusted for age, nulliparity, and adiponectin).

### 3.5 Predictive value of ZJU for the GDM

As shown in Figure 3 and Table 5, ROC curves evaluated the diagnostic performance of the ZJU index for GDM. The ZJU index exhibited superior predictive ability for GDM, with an area under the curve (AUC) of 0.802 (95% CI: 0.72–0.88), significantly outperforming single biomarkers: TG (AUC = 0.781, 95% CI: 0.70–0.86), FPG (AUC = 0.659, 95% CI: 0.56–0.76), ALT/AST (AUC = 0.648, 95% CI: 0.55–0.75), and HDL (AUC = 0.603, 95% CI: 0.50–0.71). The optimal diagnostic cutoff for the ZJU index, identified using the Youden index method, was 33.64351, yielding a sensitivity of 0.722 and specificity of 0.7577.

**Table 5:** The AUC, cut-off point, sensitivity, and specificity of ZJU in GDM.

Variable	AUC (95% CI)	Cut-off point	Specificity	Sensitivity
ZJU	0.802 (0.72–0.88)	33.64351	0.7577	0.7222
FPG	0.659 (0.56–0.76)	91	0.9581	0.3056
ALT/AST	0.648 (0.55–0.75)	0.913	0.8124	0.4722
HOMA-IR	0.766 (0.68–0.85)	2.8	0.878	0.5833
TG	0.781 (0.70–0.86)	122	0.6448	0.8333
BMI	0.747 (0.65–0.84)	23.71	0.7796	0.6667
HDL	0.603 (0.50–0.71)	49	0.8852	0.3611

AUC, area under the curve; CI, confidence interval; BMI, body mass index; TG, triglyceride; HDL, high-density lipoprotein cholesterol; FPG, fasting plasma glucose; ALT, alanine aminotransferase; AST, aspartate aminotransferase; HOMA-IR, homeostatic model assessment of insulin resistance; GDM, gestational diabetes mellitus.

### 3.6 Sensitivity analysis

The results of sensitivity analyses are detailed in Supplementary Table S3. First, multivariable regression on PSM data (baseline characteristics of the matched cohort in Supplementary Table S4) demonstrated a consistent association: ZJU index was significantly associated with GDM risk (OR = 1.22, 95% CI: 1.12–1.32, *p* < 0.0001). Subsequently, adding NAFLD as a covariate in the fully adjusted model did not alter the stability of the association (OR = 1.18, 95% CI: 1.08–1.28, *p* = 0.0003). Additionally, restricting the analysis to pregnant women with normal liver function (ALT ≤40 U/L, AST ≤40 U/L, GGT ≤50 U/L) yielded a stable effect estimate (OR = 1.22, 95% CI: 1.12–1.33, *p* < 0.0001). Finally, to address potential bias from small sample size, Firth penalized regression was applied, confirming the association (OR = 1.21, 95% CI: 1.12–1.32, *p* < 0.0001). Collectively, these multiple sensitivity analyses consistently demonstrated the robust association between the ZJU index and GDM risk (all *p* < 0.05).

As shown in Supplementary Table S5, the *E*-value for the ZJU index-GDM association was 1.74, indicating that an unmeasured confounder would need to have a risk ratio of at least 1.74 (relative to both the exposure and outcome) to fully explain away the observed effect. This suggests that the association is unlikely to be substantially influenced by unknown or unmeasured confounding factors, thereby supporting the reliability of our findings.

## 4 Discussion

In this prospective cohort study involving 585 Korean women, the ZJU index was significantly and positively associated with GDM (OR = 1.22, 95% CI: 1.13–1.32), exhibiting a linear dose-response relationship. Multiple sensitivity analyses supported the robustness of these findings. The ZJU index demonstrated high predictive performance for GDM, with AUC of 0.802. Collectively, these results suggest that the ZJU index serves as an effective predictive marker for GDM.

The association between liver enzymes, lipids, and subsequent GDM has been controversial in prior research. Our study found no independent association between individual liver enzyme markers and GDM in early pregnancy, while among lipid profiles, only TG (OR = 1.02, 95% CI: 1.01–1.02, *p* < 0.0001) and HDL (OR = 0.96, 95% CI: 0.94–0.99, *p* = 0.0079) showed correlations. These findings are directionally consistent with but have smaller effect sizes than those of retrospective studies (TG: 1.25 vs. 1.02), potentially due to differences in study design (recall bias in retrospective cohorts) or cultural, dietary, and environmental variations between countries (29). Notably, while individual liver enzyme levels were not directly associated with GDM risk, the ALT/AST ratio exhibited significant correlations (OR = 2.80, 95% CI: 1.04–7.56), likely because this ratio better identifies hepatic steatosis than single enzyme markers (30). This aligns with similar studies (31), though the lower effect size reported in a Chinese two-center study (OR = 1.60, 95% CI: 1.10–2.34) may relate to differences in gestational age at measurement (10–14 weeks vs. 8–12 weeks) and unmeasured residual confounding. The complex pathogenesis of GDM makes it difficult for a single liver enzyme index to fully reflect its pathogenesis, which is consistent with previous studies (32–34). Therefore, the establishment of a comprehensive monitoring system that includes multiple biomarkers is crucial to accurately assess the risk of GDM.

Although the precise pathophysiological mechanisms of GDM are not fully understood, extensive evidence links hepatic dysfunction and lipid metabolism abnormalities to GDM pathogenesis (17, 35–37). Recent studies suggest that lipid metabolites play a pivotal role in mediating the complex relationship between HSI, a reliable biomarker for non-alcoholic fatty liver disease, and GDM (38). Obesity is identified as a contributing factor within the link between lipids and GDM (39). In light of these findings, this study hypothesized that the ZJU index was associated with the occurrence of GDM.

As shown in previous studies, there was a correlation between ZJU nonalcoholic fatty liver disease, T2DM, as well as insulin resistance (19–21, 40). Large cohort studies have shown that the ZJU index is a reliable tool for identifying NAFLD (19). A cross-sectional study in China including 3,329 participants demonstrated that the ZJU index served as a robust indicator for identifying insulin resistance (IR) in the general Chinese population, with the risk of IR significantly elevated in the highest quartile of the index (20). In a Japanese prospective cohort study of 15,464 participants, the ZJU index was positively associated with incident diabetes in the general population (21). However, the utility of the ZJU index in pregnant women remains underexplored. To the best of our knowledge, this study is among the first to investigate the associations of the ZJU index with NAFLD and GDM during pregnancy.

As demonstrated in previous studies, the ZJU index has been shown to possess a significantly higher predictive efficacy for NAFLD than a single biomarker (AUC = 0.823) (41). The present study further confirmed its equally strong predictive ability for GDM (AUC = 0.802). Despite the findings of numerous studies indicating that TG, HDL, FPG, ALT/AST ratio, BMI and HOMA-IR are independently associated with the risk of GDM (31, 42–45), the results of the ROC analysis demonstrated that the predictive efficacy of the ZJU index was significantly superior to that of the aforementioned indicators (AUC range: 0.603–0.781). It is noteworthy that the risk of GDM was found to be considerably elevated at a ZJU index threshold greater than 33.64351 in early pregnancy, indicating the necessity for early clinical intervention.

The ZJU index combines FPG, lipids, liver enzymes and BMI. It is a representative marker of irregular liver metabolism. Although the relationship between the ZJU index and GDM is unknown, the relationship with insulin resistance is not difficult to speculate. The liver is critical for the maintenance of glucose homeostasis and insulin resistance mechanisms (46). Abnormal hepatic metabolism drives increased hepatic TG synthesis and accumulation of unbound fatty acids and toxic lipids, which disrupt insulin signaling in pancreatic β cells and promote abnormal glucagon secretion (47). Concurrently, abnormal weight further aggravates the excessive expansion of fat reserves and the accumulation of fat in atypical areas, inducing lipotoxic effects that impair organelle function. This process is accompanied by massive reactive oxygen species release and intensified inflammatory responses, triggering systemic inflammation that disrupts insulin signaling pathway efficacy and culminates in insulin resistance (21, 48, 49). Furthermore, hepatic metabolic dysfunction upregulates ALT activity, sustaining inappropriate gluconeogenesis; in this state, insulin fails to suppress hepatic gluconeogenesis effectively, exacerbating insulin resistance and inducing hyperglycemia and hyperinsulinemia (50, 51). During pregnancy, hyperglycemia induces insulin resistance via oxidative stress-mediated cellular dysfunction (52). Therefore, it is reasonable to assume that the ZJU index and GDM are closely related and may be a risk factor for GDM in early pregnancy.

Our study found that the ZJU index was closely related to non-alcoholic fatty liver disease and GDM. This finding, that identifying women at risk for NAFLD and GDM early in pregnancy provides a simple and effective indicator with high clinical utility. Specifically, the ZJU index demonstrates its superiority over single indices in predicting GDM risk, thus supporting its incorporation into routine screening processes to significantly enhance the accuracy of identifying female patients with GDM. Additionally, the ZJU index, with its accessibility and cost-effectiveness, facilitates broader application in clinical practice and enhances its utility in clinical decision-making. The ZJU index exhibits considerable predictive efficacy in the early stages of pregnancy, which is crucial for implementing personalized early intervention treatments. Early identification and treatment measures have been shown to effectively prevent the progression of GDM, reduce complications during pregnancy and postpartum, thereby promoting women’s health.

In conclusion, this study presents two principal findings. First, the correlation between single liver enzymes and lipids and GDM is limited; thus, comprehensive prediction through multiple biomarkers is crucial. Second, the ZJU index not only correlates with NAFLD during early pregnancy but also demonstrates robust predictive efficacy for GDM, thereby providing a novel strategy for early prevention, precise diagnosis, and personalized clinical interventions.

One key strength of our study is its prospective design. We innovatively investigated the association between the ZJU index and the risk of GDM in early pregnancy. Further studies are necessary to confirm and validate the potential role of the ZJU index in predicting GDM during early pregnancy and to explore the underlying biological mechanisms. However, several limitations are acknowledged. First, this study demonstrated only the association between the ZJU index and GDM without establishing a causal relationship. Additionally, this study’s sample is limited to Korean women, and the generalizability of the findings to other racial/regional populations may be restricted due to the regional heterogeneity in GDM incidence and differences in metabolic characteristics and environmental factors across populations (2). Although the ZJU index has been validated in East Asian populations, its predictive efficacy for GDM in non-Asian populations remains unclear, and relevant thresholds require further calibration. Furthermore, our analysis was confined to the first trimester (10–14 weeks of gestation), limiting the evaluation of its predictive value in mid-pregnancy and the postpartum period. As a secondary analysis, inherent limitations are present despite adjustments for major covariates. Specifically, the inability to prospectively define variables led to potential residual confounding from unmeasured factors, such as family history of diabetes, gestational age, medication use, lifestyle behaviors, and genetic predisposition. To assess the effect of these unmeasured confounders, the E value was calculated, revealing a relatively weak effect on our conclusions. Additionally, the diagnosis of NAFLD in this study primarily relied on liver ultrasonography, not histopathological examination. Notably, histological confirmation of NAFLD is challenging in asymptomatic pregnant individuals due to the invasive nature of tissue sampling. Finally, using a single baseline ZJU Index assessment in early pregnancy potentially introduces measurement errors and misses dynamic changes across gestation.

Future research will focus on conducting comprehensive dynamic monitoring studies that provide in-depth insights into physiological changes throughout pregnancy. Additionally, efforts are needed to validate the generalizability of the ZJU Index across diverse racial, geographical, and clinical populations and facilitate its integration into evidence-based GDM screening algorithms.

## 5 Conclusion

Our findings demonstrate that an elevated ZJU index in early pregnancy is significantly associated with GDM, with extensive sensitivity analyses supporting the robustness of these results. This index provides a novel approach to GDM’s early screening and monitoring. Prospective validation across multicenter cohorts with diverse ethnic populations and interventional trials stratified by ZJU index thresholds are imperative to establish generalizability and evaluate its clinical utility in informing personalized GDM prevention strategies.

## Data Availability

Publicly available datasets were analyzed in this study. This data can be found here: the dataset supporting the conclusions of this article is available in the (PLoS One) repository (which may be accessed at: https://journals.plos.org/plosone/).

## Glossary

GDM: Gestational diabetes mellitus
CVD: Cardiovascular disease
T2DM: Type 2 diabetes mellitus
ALT: Alanine aminotransferase
AST: Aspartate aminotransferase
TG: Triglyceride
NAFLD: Nonalcoholic fatty liver disease
FPG: Fasting plasma glucose
BMI: Body mass index
RCS: Restricted cubic splines
ROC: Receiver operating characteristic
AUC: Area under the curve
HOMA-IR: Homeostatic model assessment for insulin resistance
PSM: Propensity score matching
SMD: Standardized mean differences
DAGs: Directed acyclic graphs
VIF: Variance inflation factor
IR: Insulin resistance
OR: Odds ratio
CI: Confidence interval
HDL: High-density lipoprotein cholesterol
LDL: Low-density lipoprotein cholesterol
TC: Total cholesterol
FFA: Free fatty acid
