# Supplementary material for: ZJU index as a predictive biomarker of gestational diabetes mellitus: a prospective cohort analysis
Source: Front Nutr. 2025 Jun 13;12:1570771. doi: 10.3389/fnut.2025.1570771 (PMC12202635; doi:10.3389/fnut.2025.1570771)
Supplement: Supplementary file 1 [file Table_1.docx]

**Supplementary Materials**

**ZJU index as a predictive biomarker of gestational diabetes mellitus: a prospective cohort analysis.**

Ziyi Xua, Xuewei Lib , Hui Wangb,Liuyang Xua,Changhui Lia*

**Table S1 Variance Inflation Factor.**

**Table S2. Box-Tidwell Test for Assessment of Linear Relationship Between ZJU and GDM Risk**

**Table S3. Association between ZJU and GDM in multivariable logistic regression with sensitivity analysis**

**Table S4. Comparison of the two sets of statistics after adjustment**

**Table S5. The possibility for unobserved confounding between ZJU and the risk of GDM by calculating E values.**

**Figure S1.** Directed acyclic graphs

**Table S1 Variance Inflation Factor**

| Variables | VIF |
| --- | --- |
| ZJU | 1.09 |
| ADIPONECTIN | 1.07 |
| AGE | 1.09 |
| PARITY | 1.09 |

**Table S2. Box-Tidwell Test for Assessment of Linear Relationship Between ZJU and GDM Risk**

| Term | Coefficient | P_value |
| --- | --- | --- |
| ZJU*ln(ZJU) | 0.023 | 0.96 |

**Table S3. Association between ZJU and GDM in multivariable logistic regression with sensitivity analysis**

| **Variable** | **OR (95%CI), P-value** | | | |
| --- | --- | --- | --- | --- |
|  | **Model 1** | **Model 2** | **Model 3** | **Model 4** |
| Continuous |  |  |  |  |
| ZJU | 1.22 (1.12, 1.32) <0.0001 | 1.18 (1.08, 1.28) 0.0003 | 1.22 (1.12, 1.33) <0.0001 | 1.21(1.12,1.32)<0.0001 |
| Categories |  |  |  |  |
| Q1 | Ref. | Ref. | Ref. |  |
| Q2 | 2.08 (0.39, 11.24) 0.394 | 3.59 (0.73, 17.68) 0.1160 | 3.84 (0.79, 18.72) 0.0960 | 3.08(0.82,16.65)0.098 |
| Q3 | 6.71 (1.50, 29.95)0.013 | 5.22 (1.15, 23.63) 0.0320 | 7.23 (1.64, 31.84) 0.0090 | 6.24(1.94,31.53)0.001 |

OR = Odds Ratio, CI = Confidence Interval

Model 1: Sensitivity analysis after PSM.Adjusted for Age; Nulliparity; Adiponectin.

Model 2: Sensitivity analysis with NAFLD included as a covariate.Adjusted for Age; Nulliparity; NAFLD; Adiponectin.

Model 3: Sensitivity analysis restricted to individuals with ALT ≤ 40 U/L, AST ≤ 40 U/L, and GGT ≤ 50 U/L.Adjusted for Age; Nulliparity; Adiponectin.

Model 4: Firth-corrected regression.Adjusted for Age; Nulliparity; Adiponectin.

### Table S4. Comparison of the two sets of statistics after adjustment

| **Variables** | **Before Matching** | | | **After Matching** | | |
| --- | --- | --- | --- | --- | --- | --- |
|  | **ZJU< 31.19** | **ZJU≥ 31.19** | **SMD** | **ZJU< 31.19** | **ZJU≥ 31.19** | **SMD** |
| **n** | 292 | 293 |  | 275 | 275 |  |
| Age, Mean ± SD | 31.88 ± 3.63 | 32.27 ± 3.91 | 0.099 | 31.85 ± 3.56 | 31.83 ± 3.75 | -0.007 |
| Nulliparity(%) |  |  |  |  |  |  |
| No | 158 (54.11) | 149 (50.85) | -0.065 | 129 (53.31) | 122 (50.41) | -0.058 |
| Yes | 134 (45.89) | 144 (49.15) | 0.065 | 113 (46.69) | 120 (49.59) | 0.058 |

SMD=Standardized Mean Difference

**Table S5. The possibility for unobserved confounding between ZJU and the risk of GDM by calculating E values.**

| Calculate E-value | relative risk of exposure-confounder | relative risk confounder-outcome |
| --- | --- | --- |
| 1.74 | 1.46 | 2.34 |

**Figure S1. Directed acyclic graphs**


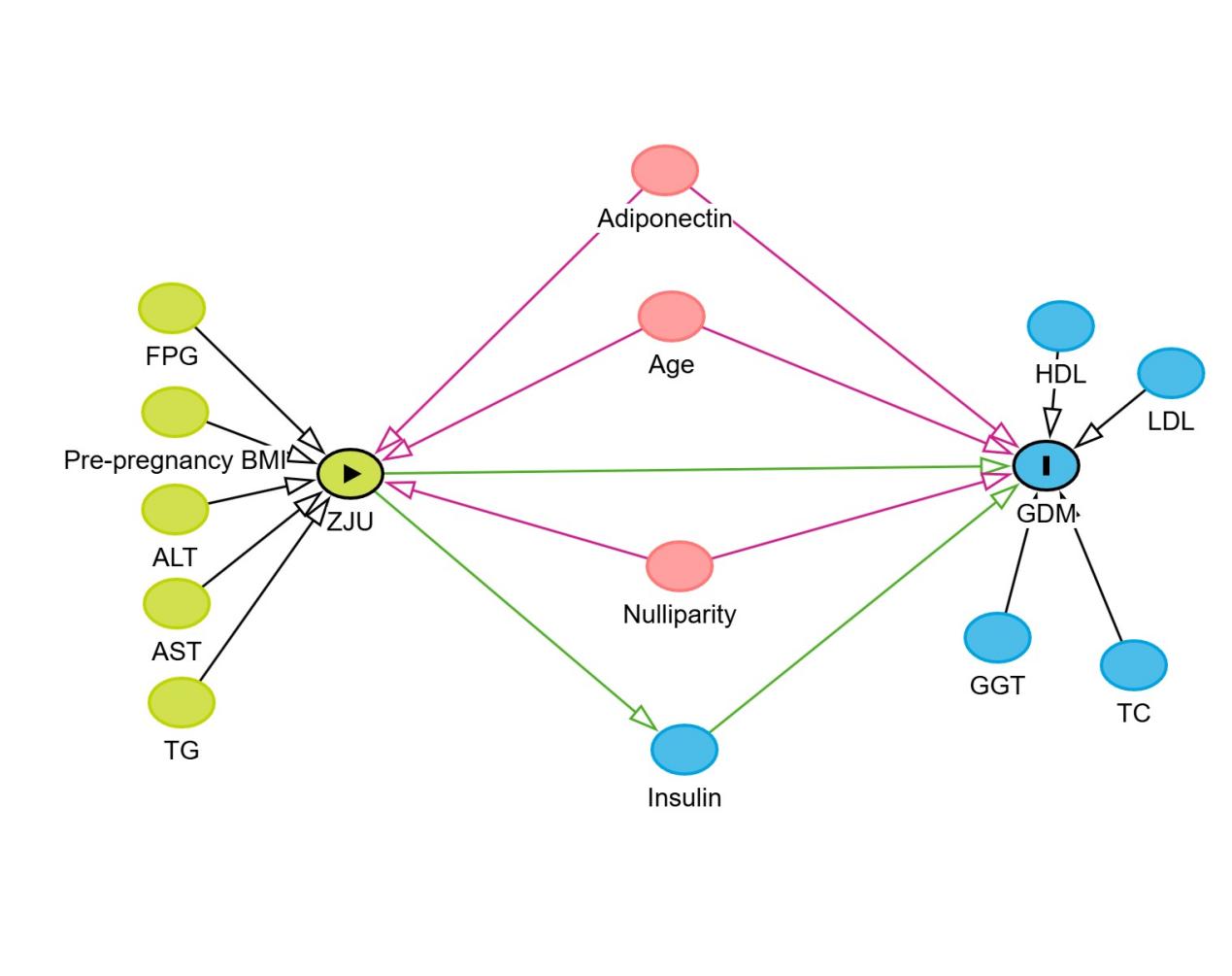


Triglyceride(TG),total cholesterol (TC), high-density lipoprotein cholesterol(HDL), low-density lipoprotein cholesterol (LDL),Pre-pregnancy body mass index (BMI) ,fasting blood glucose(FBG),Alanine Aminotransferase (ALT), Gamma-glutamyltransferase (GGT), Aspartate Aminotransferase (AST)and Gestational diabetes mellitus(GDM).
